# Promoting Cas9 degradation reduces mosaic mutations in non-human primate embryos

**DOI:** 10.1038/srep42081

**Published:** 2017-02-03

**Authors:** Zhuchi Tu, Weili Yang, Sen Yan, An Yin, Jinquan Gao, Xudong Liu, Yinghui Zheng, Jiezhao Zheng, Zhujun Li, Su Yang, Shihua Li, Xiangyu Guo, Xiao-Jiang Li

**Affiliations:** 1 State Key Laboratory of Molecular Developmental Biology, Institute of Genetics and Developmental Biology, Chinese Academy of Sciences, Beijing, 100101 China; 2 Guangdong-Hongkong-Macau Institute of CNS Regeneration, Ministry of Education CNS Regeneration Collaborative Joint Laboratory, Jinan University, Guangzhou, 510632, China; 3 Yuanxi Biotech Inc., Guangzhou, 510663 China; 4 Department of Human Genetics, Emory University School of Medicine, Atlanta, GA 30322, USA.

## Abstract

CRISPR-Cas9 is a powerful new tool for genome editing, but this technique creates mosaic mutations that affect the efficiency and precision of its ability to edit the genome. Reducing mosaic mutations is particularly important for gene therapy and precision genome editing. Although the mechanisms underlying the CRSIPR/Cas9-mediated mosaic mutations remain elusive, the prolonged expression and activity of Cas9 in embryos could contribute to mosaicism in DNA mutations. Here we report that tagging Cas9 with ubiquitin-proteasomal degradation signals can facilitate the degradation of Cas9 in non-human primate embryos. Using embryo-splitting approach, we found that shortening the half-life of Cas9 in fertilized zygotes reduces mosaic mutations and increases its ability to modify genomes in non-human primate embryos. Also, injection of modified Cas9 in one-cell embryos leads to live monkeys with the targeted gene modifications. Our findings suggest that modifying Cas9 activity can be an effective strategy to enhance precision genome editing.

CRISPR-Cas9 combines the type II bacterial clustered, regularly interspaced, palindromic repeats (CRISPR)-associated (Cas) system and a single guide RNA (gRNA), which targets Cas9 to genomic regions complementary to the gRNA^1^. Double-stranded DNA breaks at the targeted locus are generated by Cas9 digestion and repaired by non-homologous end-joining (NHEJ) or homology-directed repair (HDR), resulting in indel mutations^2^,^3^. Thus, CRISPR-Cas9-mediated genomic mutations can be used to create animal models that mimic genetic mutations in human diseases. The ability of CRISPR-Cas9 to modify genomes in embryos opens up a new avenue to generate genetically modified animal models without the need to use embryonic stem cells^4^,^5^. Indeed, CRISPR-Cas9 has been used to target genes in large animals whose embryonic stem cells are not available for genetic manipulation. For example, CRISPR-Cas9 was recently used to directly modify embryonic genomes and create animal models of human diseases in large animals, including rabbits^6^, dogs^7^, pigs^8^,^9^,^10^, and nonhuman primates^11^,^12^.

Unfortunately, however, this technology also creates mosaic mutations in embryos, resulting in different mutations in different types of cells in the same animal^5^,^13^,^14^. For example, mosaic mutations are quite extensive in zebra fishes that were targeted by CRISPR/Cas9^15^,^16^,^17^. For small animals with short breeding times, mosaic mutations can be diluted over generations, and specific mutations in DNA can be obtained by crossing animals for 4–5 generations. In contrast, for large animals like nonhuman primates, whose sexual maturation requires 4–5 years, mosaic mutations can be an obstacle to obtaining genetically modified animals that can faithfully mimic genetic mutations in human patients. More importantly, mosaicism can significantly affect the precision of gene therapy when CRISPR-Cas9 technology is used.

CRISPR-Cas9-mediated mosaic mutations likely result from random DNA breaks and repairs, but identifying the mechanisms behind this mosaic DNA mutation phenomenon has proved a challenge. One possible explanation is that the prolonged expression of Cas9 in embryos could continuously generate DNA breaks, thereby increasing the mosaic mutation rate. For example, Cas9 mRNA may segregate into late embryo stages, such as the 4-cell to morula stages, to act on targeted DNAs, thus creating mosaicism in targeting. Alternatively, the degree of mutation induction by the CRISPR/Cas9 system might be different in each blastomere of 2-cell- to 4-cell-stage embryos, leading to the generation of offspring cells with various mutation types. It is reasonable to speculate that Cas9 synthesized from the injected mRNA remains active throughout several cell divisions in early embryo development, which may trigger different mutations in different blastomeres, leading to mosaic embryos.

Based on the above hypotheses, we wondered whether shortening the half-life of Cas9 would reduce mosaic mutations in early embryos. To explore this, we tagged the ubiquitin-proteasome degradation signal to the N-terminus of Cas9 and found that this could reduce the half-life of Cas9 without affecting its DNA cleavage activity. This modified Cas9 apparently decreases mosaicism in DNA mutations in monkey embryos. More importantly, using this modified Cas9, we generated a live monkey with the ASPM mutations. Our findings therefore suggest that modifying Cas9 stability can be an effective strategy to enhance precision genome editing.

## Results

### Tagging UPS Degradation Signals to Cas9 to Reduce Its Half-Life

**Figure 1:**
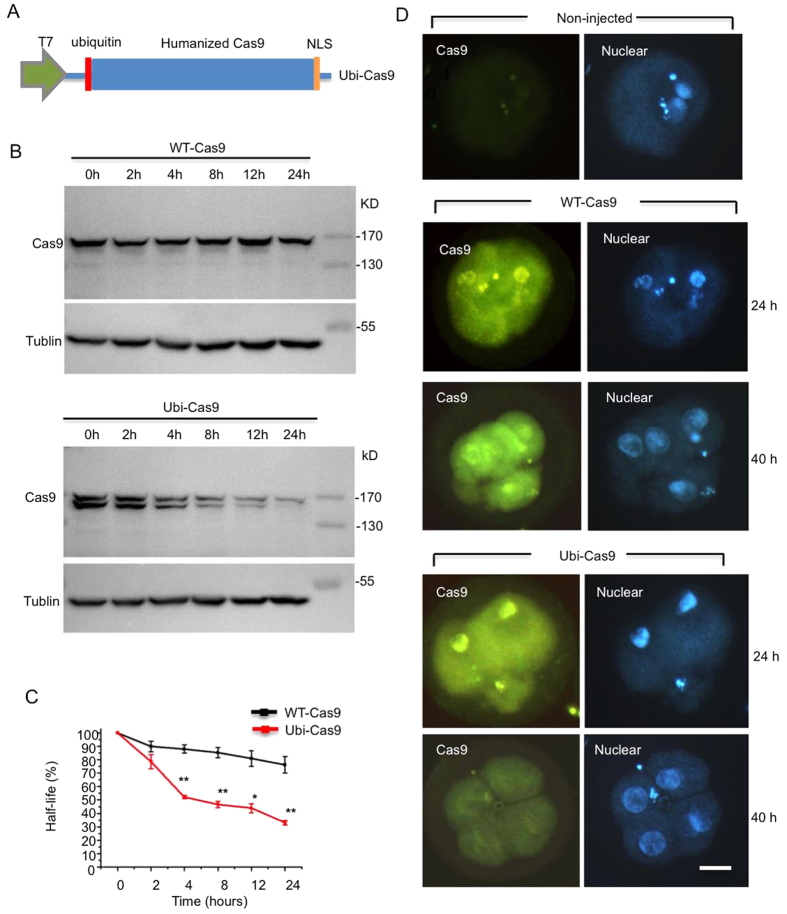
Targeting Ubiquitin to Cas9 to Increase Its Degradation. **(A)** Schematic of the Ubiquitin-Cas9 (Ubi-Cas9) vector for the expression of Cas9 with short half-life. Red box indicates the inserted ubiquitin-targeting signal that is tagged to the N-terminus of Cas9. Green box indicates T7 promoter. The C-terminus of Cas9 carries the nuclear localization signal (NLS). (**B**) Western blot analysis for comparing the half-life of WT-Cas9 and Ubi-Cas9 in transfected HEK293 cells that were treated with 50 μM cycloheximide for different times (0, 4, 8, 12, 24 h) to inhibit protein synthesis. The relative protein levels of WT-Cas9 and Ubi-Cas9 were assessed by densitometric analysis of their bands on western blots, and the value at 0 h was considered 100% (mean ± SEM; n = 3, *p < 0.05; **p < 0.01). Images of full-length gels are seen in supplemental information. (**C**) Immunocytochemical assays showing the rapid degradation of Ubi-Cas9 in monkey embryos after injection of Ubi-Cas9 mRNA for 24 h or 40 h. WT-Cas9 mRNA injection served as controls. Embryos were stained with anti-Cas9 (green) and DAPI (blue) for nuclear staining. Note that more Ubi-Cas9 is located in the nucleus than the cytoplasm. Scale bar: 20 μm.

To explore whether reducing the half-life of Cas9 could reduce mosaic mutations, we added ubiquitin-targeting signal (Ubiquitin-**R**GKEQKLISEEDL-CAS9) to the N-terminus of Cas9 (Fig. 1A), as this targeting can facilitate protein degradation by the proteasome^18^. After expressing this modified Ubi-Cas9 protein in HEK293 cells via transfection, we compared its half-life with wild-type Cas9 (WT-Cas9) in transfected HEK293 cells that were treated with cycloheximide (CHX), an inhibitor of protein synthesis, for different time periods. Western blot results clearly show that Ubi-Cas9 is degraded much faster than WT-Cas9 (Fig. 1B,C). Since it is important to know whether this tagging affects the activity of Cas9 to cut DNA, we next generated His-tagged WT-Cas9 and Ubi-Cas9 to compare their abilities to cut Pink1 and Parkin DNAs, mutations in which cause Parkinson’s disease^19^ (Figure S1A,B). Using different doses of these purified Cas9 proteins to cut Pink1 DNA in the presence of its gRNA, we found no difference in the abilities of WT-Cas9 and Ubi-Cas9 to digest Pink1 DNA (Figure S1B,C). We also performed time-course studies of Cas9’s ability to cut different Pink1 and Parkin DNAs. Although *in vitro* DNA digestion efficiency by Cas9 is dependent on DNA sequences, we found no difference in the abilities of WT-Cas9 and Ubi-Cas9 to digest different DNAs (Figure S1C,D). Furthermore, Ubi-Cas9 could still generate DNA mutations with gRNA when both were transfected into HEK293 cells to target the Parkin and Pink1 genes (Figure S2).

### Tagging UPS Degradation Signals Does Not Affect the Ability of Cas9 to Cut DNAs In Monkey Embryos

We next compared the gene-targeting efficiency of WT-Cas9 and Ubi-Cas9 on embryos of cynomolgus monkeys using a method described in our previous study^12^. We collected MII oocytes from female cynomolgus monkeys after superovulation and used them for intracytoplasmic sperm injection (ICSI). We first wanted to confirm that Ubi-Cas9 has a shorter half-life than WT-Cas9 in monkey embryos. After the fertilized eggs were intracellularly injected with mRNAs of WT-Cas9 or Ubi-Cas9 (200 ng/μl), we collected 2-cell embryos at 24 h and 4-cell embryos at 40 h. Immunofluorescent staining clearly showed that Ubi-Cas9 expression was virtually the same as WT-Cas9 expression in 2-cell embryos but rapidly declined in 4-cell embryos, whereas WT-Cas9 continued to be maintained at a high level (Fig. 1D).

**Figure 2:**
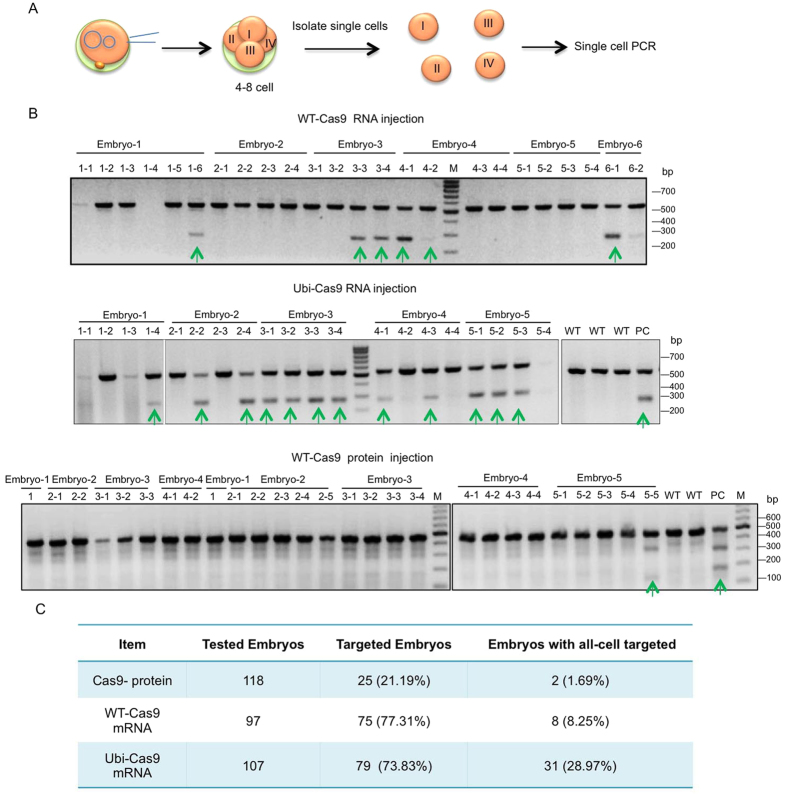
Comparison of Pink1 DNA Targeting by Injection of Cas9 mRNA and Cas9 Protein. Identification of Pink1 DNA mutations by T7E1 assay in monkey embryos injected with WT-Cas9 or Ubi-Cas9 mRNA (**A**) or purified WT-Cas9 protein (**B**). Representative DNA gel images showing PCR results of single cells from different embryos. Arrow indicates DNA mutation in a single cell from 8-cell embryos. PC is a positive control from embryos injected with Cas9 mRNA and gRNA for Pink1 targeting. M: Molecular markers. **(C)** Comparison of targeting rates of injected Cas9 protein, WT-Cas9 mRNA, and Ubi-Cas9 mRNA in monkey embryos. The numbers of embryos with targeted gene mutations or embryos containing mutations in all cells are presented. Embryos at the 4- and 8-cell stages were used, and data were obtained from six experiments.

Next we tested the targeting efficiency of Ubi-Cas9 by co-injecting gRNAs to target the gene for Pink1 and abnormal spindle-like microcephaly-associated protein (ASPM), the absence of which causes autosomal recessive primary microcephaly (MCPH5)^20^. Since the direct injection of Cas9 protein is known to yield DNA mutations^21^,^22^,^23^ and since Cas9 protein may have a shorter half-life than Cas9 mRNA due to proteolysis, we also directly injected WT-Cas9 protein for comparison. After injection of gRNAs with WT-Cas9 protein, WT-Cas9 mRNA, or Ubi-Cas9 mRNA, we collected embryos at different developmental stages (4 to 8 cells). Since analysis of whole-embryo DNA is problematic for evaluating mosaic mutations and targeting efficiency, we isolated single cells for PCR from 4- or 8-cell embryos (Fig. 2A). PCR analysis revealed that Ubi-Cas9 and WT-Cas9 mRNA injections had similar embryo targeting rates (73.83% versus 77.31%) when counting the number of embryos carrying mutations. On the other hand, direct injection of wild type Cas9 protein, either our purified Cas9 or commercially obtained Cas9, yielded the lowest rate (21.19% for embryos with mutations) (Fig. 2B,C). Thus, unlike cultured cells and mice in which injected Cas9 proteins were found to yield a high targeting rate^21^,^22^,^23^, primate embryos may have unknown mechanisms that reduce the targeting efficiency of injected Cas9 proteins. It was also recently reported that direct expression of Cas9 protein in early-stage mouse zygotes could reduce mosaic mutations^24^. Thus, species difference in embryos, embryonic stages, and the quality of Cas9 proteins and mRNA could account for the discrepancy of mosaic mutations mediated by the injected Cas9 protein in different studies.

Although injection of Cas9 mRNA yielded a higher rate of targeting in whole embryos than direct injection of Cas9 protein, we noticed that not all cells in the same embryos are targeted by WT-Cas9. We then compared the targeting rate in separated cells from the same embryos via single-cell PCR. When examining mutations in all individual cells in a whole embryo, we found that 28.97% of embryos injected with Ubi-Cas9 mRNA contained mutations in all cells, whereas only 8.25% of embryos injected with WT-Cas9 mRNA and 1.69% of embryos injected with WT-Cas9 protein showed mutations in all cells (Fig. 2C). This difference suggests that Ubi-Cas9 RNA injection can lead to a higher rate of homogenous targeting in different individual cells in early embryos.

### Shortening Cas9 Half-Life Reduces Mosaic Mutations in Monkey Embryos

**Figure 3:**
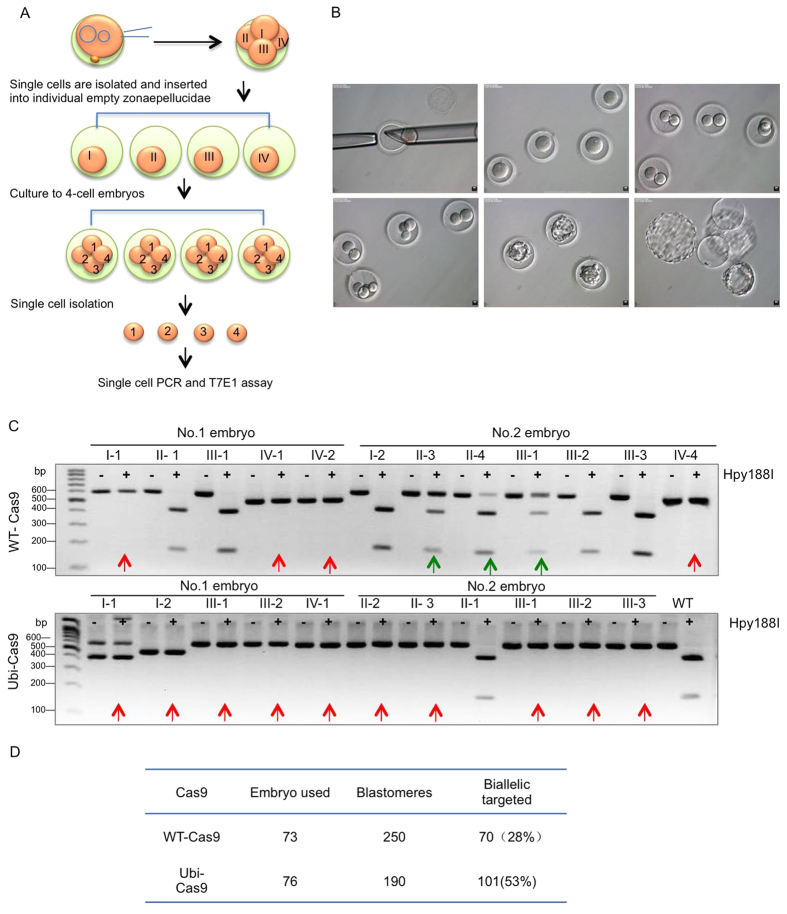
Use of Embryo Splitting to Assess Ubi-Cas9 Targeting Efficiency. **(A)** Embryo splitting at the 4-cell stage to separate single cells (I, II, III, and IV), which were placed into individual empty zonae pellucidae to further develop to divided embryos that consist of 4 cells (1, 2, 3, and 4). Single cells (1, 2, 3, and 4) were then isolated and used for PCR analysis. **(B)** Photographs of development of divided monkey embryos: a single cell isolated from 4-cell embryos was transferred into an empty zona pellucida, and then developed to 2- or 4-cell or hatched blastocyst. (**C**) Hpy188I digestion of the targeted Pink1 gene showing that Ubi-Cas9 mRNA injection yielded a high rate of homogenous mutations in monkey embryos. PCR products of single cells (1, 2, 3, 4) from divided embryos were analyzed. For Hpy188I digestion, red arrows indicate biallelic mutations, whereas green arrows indicate a single allele mutation. Images of full-length gels are seen in supplemental information. **(D)** In cultured blastomeres that were isolated from embryos injected with Ubi-Cas9 or WT-Cas9, Ubi-Cas9 yielded biallelic mutations in 53% of blastomeres whereas WT-Cas9 generated bialleic mutations in 28% of balstomeres.

If Ubi-Cas9 cuts DNA at the 1- or 2-cell stage and then is unable to function because it is rapidly degraded, we expect that cells generated from the same precursor at the 1- or 2-cell stage would carry the same mutations to a larger extent than those mediated by WT-Cas9, which may continue to cut DNA to generate additional mutations after the 2-cell stage. This idea could be tested by examining the gene-targeting rate in individual cells derived from the same precursor cells at the 4-cell stage. Thus, we separated cells from 4-cell embryos, and then put each cell into individual empty zonae pellucidae for culturing (Fig. 3A). Such an embryo-splitting approach was used to obtain paired demi-blastocysts of rhesus monkey that could still lead to newborn monkeys^25^,^26^. We were able to let individually divided cells from 4-cell stage embryos continuously develop to 4 cells and blastocysts (Fig. 3B). If Ubi-Cas9 activity terminates at the 4-cell stage or earlier, the genotypes of subsequently divided offspring cells should likely be the same. Thus, after the isolated single cells from 4-cell embryos had generated offspring cells, these offspring cells were separated again for single-cell PCR analysis. The offspring cells from Ubi-Cas9 mRNA-injected precursor cells showed either no mutations or contained the same mutations, such as biallelic mutations, which could be readily identified by Hpy188I digestion of the PCR products for the targeted Pink1 region (Fig. 3C). On the other hand, the offspring cells derived from WT-Cas9 mRNA-injected precursor cells contained different types of mutations (one or two allele mutations), suggesting that WT-Cas9 may still cut DNA at or after the 4-cell stage to yield different mutations in offspring cells.

**Figure 4:**
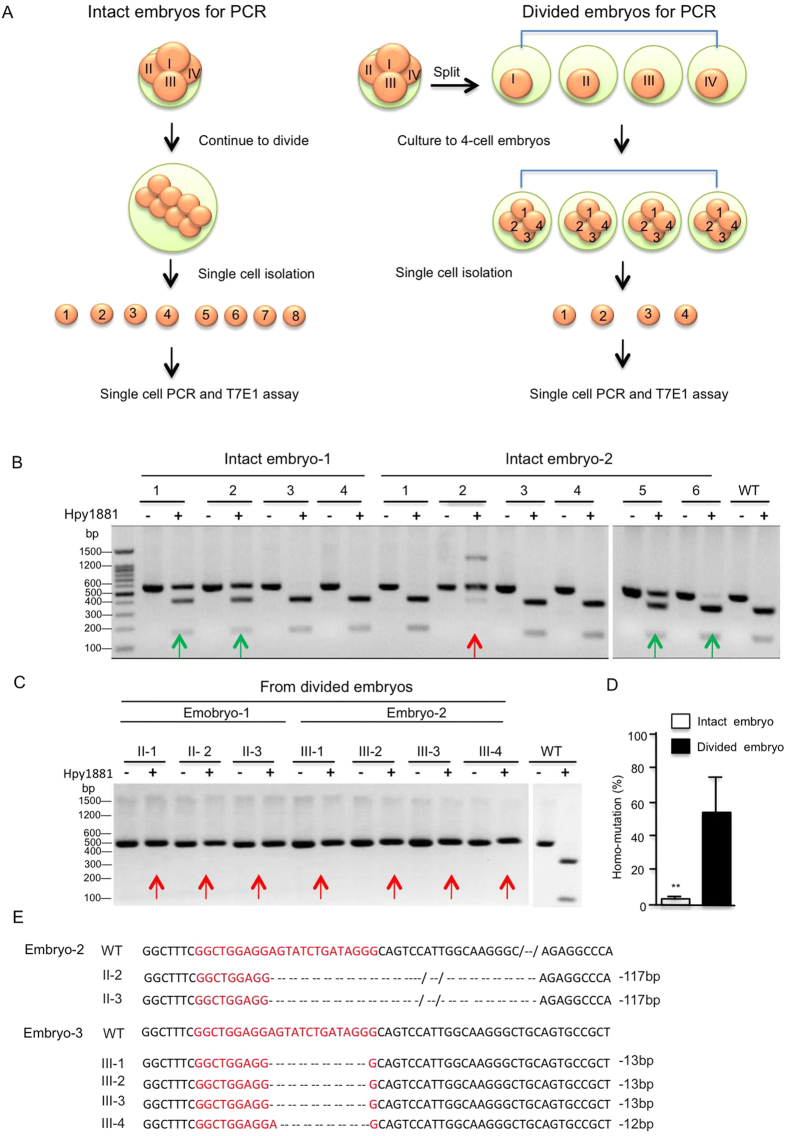
Ubi-Cas9 Targeting of Pink1 Gene. (**A**) A diagram showing single cell PCR using intact or divided monkey embryos. (**B,C**) PCR diagnosis of Pink1 exon 2 targeting via Ubi-Cas9 in single cells from intact embryos (**B**) or divided embryos (**C**). Gene mutations were diagnosed via digestion of PCR products by Hpy188I. Green arrows indicate one allele mutation, and red arrows indicate biallelic mutations. (**D**) Quantitative assessment of Ubi-Ca9-mediated homogenous mutations in single cells from intact embryos at the 4–8-cell stage or from divided embryos derived from 4-cell intact embryos. In this graph, 31 intact embryos were used to isolate single cells, and 13 4-cell-stage embryos were divided to single cells for further development to 37 single blastomere embryos; data are presented as mean ± SEM. **p < 0.01. (**E**) DNA sequencing verified the homogenous mutations in single cells from divided embryos injected with Ubi-Cas9 and gRNA for Pink1 exon 2.

In cultured blastomeres that were isolated from embryos injected with Ubi-Cas9 or WT-Cas9 and gRNAs for Pink1, Ubi-Cas9 yielded biallelic mutations in 53% of blastomeres whereas WT-Cas9 generated bialleic mutations in 28% of balstomeres (Fig. 3D). We then compared homogeneous DNA mutation rates via Ubi-Cas9 in single cells isolated from intact embryos or in offspring cells derived from the same single precursor cells that were divided at the 4-cell stage (divided embryos) (Fig. 4A). For cells from intact embryos, genotypes could be WT, one allele mutation (green arrows), or biallelic mutations (red arrow) (Fig. 4B). Also, in those cells derived from divided embryos, the same biallelic mutations could be seen in all cells (Fig. 4C). Statistical analysis revealed that < 3% of intact embryos (4–8-cell stage) were found to have the same genetic mutations in all cells, whereas 55.8% of divided embryos that were split at the 4-cell stage showed the same genetic mutations (Fig. 4D). DNA sequencing verified that each single cell from the divided embryos carried the same mutation for Pink1 exon 2 targeting (Fig. 4E). These results suggested that Ubi-Cas9 may terminate its activity at the 4-cell stage or earlier, such that subsequently divided single cells would have a high rate for the same genetic modifications or mutations.

### Generation of Live Monkeys with ASPM Mutations

**Figure 5:**
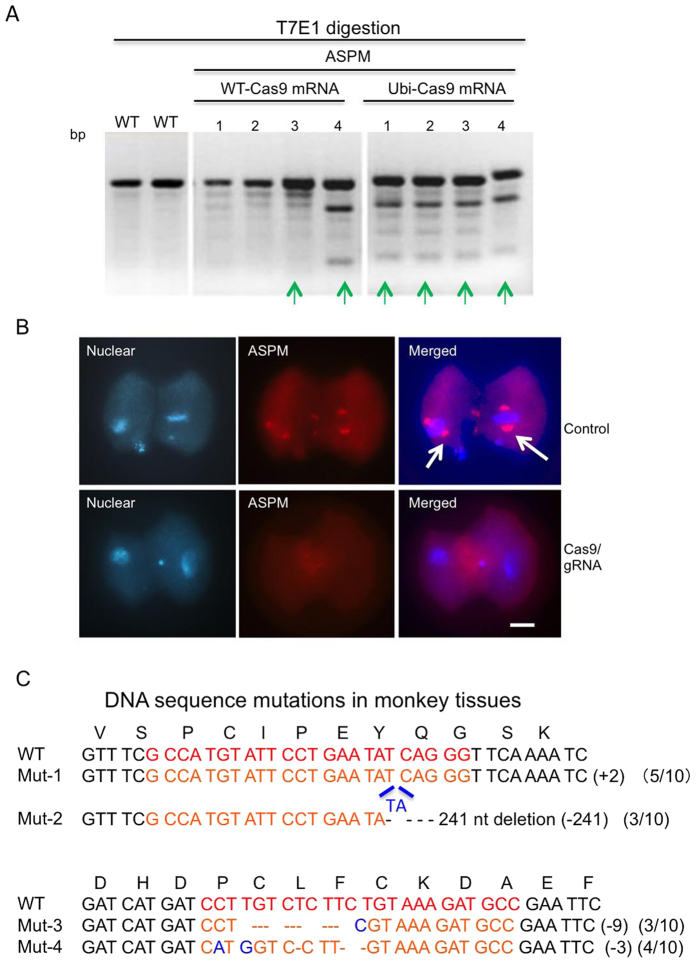
Mutations of the ASPM Gene in Monkey Tissues. (**A**) T7E1 digestion of the targeted ASPM gene in embryos injected with WT-Cas9 or Ubi-Cas9. Green arrows indicate the mutations in single cells isolated from 2-cell or 4-cell embryos. (**B**) ASPM immunofluorescent staining of 2-cell-stage monkey embryos showing that injection of Ubi-Cas9 mRNA and gRNAs for targeting ASPM can deplete the spindle localization (arrows) of ASPM. Scale bar: 20 μm. (**C**) Sequence analysis of tissue DNAs from a live monkey indicating mutations in the ASPM gene that was targeted by two gRNAs for exon 3 and exon 10. The numbers of cloned DNA for sequencing and mutated DNA clones are indicated in parentheses (mutated clones/total clones). Targeted sequence regions are indicated in red.

It is important to verify whether modified Cas9 with ubiquitin degradation signal is non-toxic to embryonic development. For ASPM targeting, we also observed a high rate of targeting by Ubi-Cas9 in single cells that were isolated from 2 or 4 cell-stage embryos (Fig. 5A). Some embryos that were injected with Ubi-Cas9 and gRNA for ASPM mutations show depletion of ASPM in the nuclear spindles of embryos, which also supports the biallelic mutations in the targeted gene (Fig. 5B). Before we could establish embryo splitting to use divided embryos that carry the homogenous mutation for generating newborn monkeys, we used intact embryos derived from zygotes injected with Ubi-Cas9 mRNAs and two gRNAs to generate ASPM mutant monkeys. We transferred 178 embryos to 47 surrogate monkeys and obtained 11 pregnant monkeys, with 6 live monkeys that were delivered healthy. Of these live monkeys, four were genotyped to carry ASPM mutations. The newborn monkeys were healthily delivered and are too young to examine their behavior. DNA sequence analysis of the monkey tissues confirmed that the two targeted regions (exons 3 and 10) in the ASPM locus contained mutations, which cause frame-shifts to yield truncated ASPM or in-frame deletions (Fig. 5C). The animals carried different mutations because they came from intact embryos injected with Ubi-Cas9 and two gRNA mRNAs. Although it would require time to examine whether the targeted monkeys develop any age-dependent phenotypes of ASPM, our studies indicate that Ubi-Cas9 targeting can generate viable animals that contain the desirable mutations in genome.

## Discussion

Although CRISPR-Cas9 has become a powerful tool to genetically modify genomes in a variety of species, this new technology has some critical technical issues, such as off-targets and mosaicism. Growing evidence indicates that off-targets can be minimized by designing more specific gRNAs and reducing Cas9 concentrations^27^,^28^,^29^,^30^, and many studies have found that the off-targets are rare if specific DNA sequences are targeted^31^,^32^,^33^. Although many studies have tried to reduce the mosaic mutations by using dual gRNAs or direct injection of Cas9 protein to target genes^24^,^34^,^35^, the mosaic issue remains to be resolved for genetically engineering non-human primate models whose long breeding periods do not allow for quick selection of specific DNA targeting via germline transmission. For the future use of CRISPR/Cas9 to correct genetic defects or for gene replacement in humans, mosaicism presents a significant hurdle to achieving the therapeutic goal of CRISPR/Cas9. Our findings suggest that reducing the half-life of Cas9 can decrease mosaicism and enable generation of a nonhuman animal model of human disease.

While the mechanism behind CRISPR-Cas9-mediated mosaicism remains elusive, the gene targeting efficiency of CRISPR/Cas9 may depend on the locus of genes, the gRNAs designed, and Cas9 activities^36^. Our findings show that shortening Cas9’s half-life can reduce mosaic mutations without affecting targeting efficiency. Many studies estimated targeting efficiency in embryos by analyzing whole embryos via PCR analysis and sequencing sub-cloned DNAs. Since this assay cannot determine the percentage of cells that carry the desired DNA mutations in embryos, we could not find a difference between WT-Cas9 and Ubi-Cas9 in terms of targeting rates when counting the embryos that show DNA mutations. However, when we separated individual cells for single-cell PCR, we found that Ubi-Cas9 could cause more embryos to have all individual cells carrying the same mutations.

After cutting DNAs by CRISPR/Cas9, DNA repair will take place by either NHEJ or HR. HR is likely to lead to re-cutting by CRISPR/Cas9 whereas NHEJ may have destroyed the targeting sites. The prolonged activity of wild type Cas9 in 2 or 4 cell-stage embryos as well as HR can contribute to mosaic DNA mutations. Although our *in vitro* experiments did not show that ubiquitin targeting signal influences the activity of Cas9 in cutting DNAs, it should be considered that ubiquitin is involved in DNA repair response by regulating protein folding^37^,^38^. Thus, it is also possible that the ubiquitin targeting at the N-terminus of Cas9 could alter its conformation *in vivo* or specifically in fertilized eggs, which might increase its ability to cut DNA at the one-cell stage, whereas WT-Cas9 may become more active after the one-cell stage. This may also explain why the overall targeting rate of Ubi-Cas9 in whole embryos is similar to WT-Cas9. Also, promoting the degradation of Cas9 may remove non-functional Cas9 from DNA more rapidly and let newly synthesized Cas9 bind to and act on DNAs more efficiently. We should also point out that whether shortening Cas9’s half-life has the same effect in early embryos from other species remains to be tested. Although these possibilities await future study, it is likely that modifying Cas9 stability or activity can reduce mosaic mutations in early embryos.

While our studies showed that Ubi-Cas9 could reduce the mosaic mutation rate, use of Ubi-Ca9 alone did not ensure that each embryo carried the same genetic mutation in individual cells in injected embryos. However, we found that embryo splitting could also help reduce mosaicism, as this splitting allows separated blastomeres to further develop to divided embryos. If Cas9, such as Ubi-Cas9, is no longer present or does not cut DNAs in the separated blastomere, some divided embryos will develop into animals that contain only a single genetic mutation or modification on the targeted locus. Such an animal model will precisely mimic a genetic mutation in human patients. Embryo splitting was reported to yield paired demi-blastocysts of rhesus monkey^25^,^26^; however, these previous studies used separated blastomeres from 2-cell-stage embryos. In our studies, we found that splitting 4-cell-stage embryos would be more likely to select divided embryos that carry the same mutation in each individual cell, perhaps because Ubi-Cas9 no longer exists after the 2-cell stage. It remains to be established that such divided embryos from 4-cell stage embryos can be implanted in surrogates for development to healthy newborn monkeys. Despite this, our studies hold great promise for the use of Ubi-Cas9 with embryo splitting to greatly increase targeting accuracy and homogeneity for genome editing.

We also proved that tagging ubiquitin to Cas9 does not cause toxicity and can still lead to the generation of live monkeys with the targeted mutations. We present the ASPM mutant monkey as an example to show the power of Ubi-Cas9 to genetically modify the genome of nonhuman primates. ASPM is evolutionarily involved in primate brain development and important for neurogenesis by participating in the normal function of the mitotic spindle^39^. Although more studies are required to investigate the pathogenesis of ASPM mutations in nonhuman primates, our findings show that modifying Cas9 stability or activity can reduce the mosaic mutations in nonhuman primate embryos. In combination with embryo splitting, the modification of Cas9 activity in early embryos will allow us to develop a strategy for generating animal models with the precise genetic mutation occurring ubiquitously in each cell, which is particularly important for gene therapy and establishing large animal models that faithfully mimic the pathology and phenotypes of human diseases.

## Methods

### Animals

Adult healthy cynomolgus monkeys (*Macaca fascicularis*) were housed in individual cages at Yuanxi Biotech Inc. Guangzhou and used in this study. All animal procedures were approved by the Institutional Animal Care and Use Committee at Yuanxi Biotech Inc. Guangzhou. All methods were performed in accordance with the relevant guidelines and regulations.

### Cas9 vectors

Cas9 plasmid (MLM3613, Plasmid #42251) was used to express spCas9 nuclease (Streptococcus pyogenes) under the control of the CMV or T7 promoter. This Cas9 carries the NLS sequences (CCCAAGAAGAAGAGGAAAGTC) at its C-terminus. The p-U6-gRNA and p-T7-gRNA expression vectors, which were used separately for HEK293 transfection and *in vitro* transcription of gRNAs, were provided by Dr. Liangxue Lai at The Guangzhou Institutes of Biomedicine and Health, CAS. gRNAs were designed based on the targeted sequences in the monkey Parkin, Pink1 and ASPM genes. Template DNAs for *in vitro* transcription were generated by PCR amplification of the gRNAs plasmids. The PCR products were purified and transcribed by mMESSAGE mMACHINE T7 kit (Ambion, AM1344) *in vitro*. The ubiquitin open reading frame and NotI-T7 promoter-ubiquitin-R-myc-NocI- was amplified by PCR from cynomolgus monkey genome DNA with the sense primer, 5′-1Not1:5′-ATCCGCGGCCGC*TAATACGACTCACTATAGG*GCCATGCAGATCTT CGTGAAGAC-3′ and the antisense primers: 3′-204 Nco1: 5′-ATCCATGGTCTTAGCATG TACCAGATCTTCTTCAGAAATAAGTTTTTGTTCTTTACCTCGCCCACCTCTGAGACGGAG-3′. Ubi-Cas9, which encodes ubiquitin and the degradation signal (GGRGKLE), and WT-Cas9 plasmids were linearized by PmeI and *in vitro* transcribed using MAXIscript T7 (Ambion, AM1312). The synthesized transcripts were added with poly-A using the *E. coli* Poly(A) Polymerase kit (NEB, M0276) and were purified using LiCl with an additional ethanol precipitation.

### Ovarian stimulation and recovery of monkey oocytes

The methods for cynomolgus monkey ovarian stimulation and oocyte recovery are similar to those for rhesus monkeys described previously^11^. Regular cycling females aged 5–8 years were subjected to follicular stimulation using twice-daily intramuscular injections of 18 IU of recombinant human FSH (rhFSH) for 8 day; then 1000 IU of human chorionic gonadotropin (HCG) were injected on day 9. Cumulus-oocyte complexes were isolated by surgery operation and aspiration 37 h post-rhCG. Follicular contents were placed in Hepes-buffered Tyrode’s albumin lactate pyruvate medium (TALP-Hepes) containing 0.3% BSA at 37 °C, supplemented with 5 IU/ml of heparin (Sigma, Inc.). Oocytes were stripped of cumulus cells with pipetting 45–60 sec and then filtered through a 70 μm cell strainer and collected in a 60 mm petri dish containing 5–7 ml of TALP-Hepes. Oocytes were picked up under a dissecting microscope to separate GV (intact germinal vesicle), metaphase I (GVB, no germinal vesicle, no polar body), metaphase II (MII, first polar body present), and other dead oocytes. Oocytes were rinsed and then transferred to 50 μl pre-equilibrated maturation medium that contained Connaught Medical Research Laboratories medium 1066 (CMRL-1066; Invitrogen Inc.) supplemented with 10% heat-inactivated fetal bovine serum (FBS), 40 μg/ml sodium pyruate, 150 μg/ml glutamine, 550 μg/ml calcium lactate under mineral oil. Immature oocytes such as GVB or GV cells were cultured in a 50 μl drop at 37.5 °C in humidified air (6% CO2) for up to 24 h.

Male cynomolgus macaques were electro-ejaculated with a current isolation stimulator (JL-C4 V2a, JIALONG, China) equipped with electrocardiographic pad electrodes for direct penile stimulation (30–50 V, 20-msec duration, 18 pulses/sec). Semen samples were collected into 15-ml tubes. Ejaculated sperm were diluted to 2 × 10^5^ in 10% polyvinylpyrrolidone (PVP) to reduce motility and were placed in a separate drop on the manipulation dish. One single sperm was aspirated from the sperm drop into the injection needle and transferred to the oocytes TALP-Hepes drop. MII oocytes were held by holding pipet on the polar body at the 6 o’clock or 12 o’clock position, then injected by the injection needle with a sperm through the zona into cytoplasm. After ICSI, oocyte were washed twice in Hamster Embryo Culture Medium 9 (HECM-9) before being transferred into a pre-equilibrated 50 μl drop of HECM-9, covered with mineral oil and incubated at 37.5 °C with 6% CO_2_ for 8–10 h. Oocytes with a second polar body and two pronuclei arising after ICSI were confirmed to have successful fertilization. Zygotes were injected with Cas9 mRNA or proteins and gRNAs, and the injected zygotes were cultured for embryo development. Embryos at 4–8 cell stages were used for transfer or divided into single blastomere for PCR. Pronuclear formation was recorded 16–20 h post-ICSI, and the progression of embryo growth was recorded daily.

### Embryo splitting

Immature GV (germinal vesicle) eggs or unfertilized oocytes were used to generate empty zonaepellucidae by XY clone laser (Hamilton Inc. USA), and empty zonaepellucidae were prepared by removing the cytoplasm via aspiration with a micropipette. A four-cell embryo was mechanically split to 4 single cells, which were aspirated into a micropipette and inserted into individual empty zonaepellucidae. The inserted single cells were then cultured in HECM-9 medium until 4 cell stage or blastocytes, those 4 cell embryos were then split again to isolate single cells for PCR analysis.

### Cas9/sgRNA injection of one-cell embryos

The zygotes were injected with Cas9 mRNA (200 ng/μl) or Cas9 protein (200 ng/μl) and gRNAs (50 ng/μl each). Cas9 proteins were generated using His-tagged Cas9 and purified using Ni-NTA Resin (Invitrogen). Commercially available and purified Cas9 (PNA, CP01) was obtained from PNA Bio.Inc. Microinjections were performed in the cytoplasm of zygotes using a Narishige microinjection system (Narishige Inc., Japan) under standard conditions. The zygotes were cultured in embryo culture medium-9 (HECM-9) containing 15% fetal calf serum (Hyclone Laboratories, SH30088.02) at 37.5 °C in 6%CO_2_. Cleaved embryos of high quality at the 4-cell stage were transferred into the oviduct of the matched recipient female monkeys. Typically, three embryos were transferred into each female. The earliest pregnancy diagnosis was performed by ultrasonography about 30–35 d after the embryo transfer. Both clinical pregnancy and number of fetuses were confirmed by fetal cardiac activity and presence of a yolk sac as detected by ultrasonography.

### Immunofluorescence staining of embryos

Ubi-Cas9 or Cas9 mRNA-injected embryos were fixed in 4% paraformaldehyde in PBS (pH 7.4) for 20 min at 4 °C. After 3 washes with PBS at room temperature, embryos were permeabilized with 0.5% Triton X-100 for 15 min, blocked in PBS supplemented with 3% BSA and 0.2% tween-20 (PBST) for 2 h, and incubated overnight at 4 °C with 1:400 rabbit anti-Cas9 antibody (Bethyl, IHC-00058). The embryos were then washed 3 times in PBST at 37 °C for 10 min each and labeled with Alexa Fluor 488 Goat Anti-mouse for 2 h at 4 °C. After another 3 washes in PBST at 37 °C for 10 min each, the embryos were co-stained with the nuclear dye DAPI (Sigma, 32670). Finally, the embryos were mounted on glass slides and examined with a laser scanning microscope (OLYMPUS IX 73, Japan).

### Cycloheximidechase assay

HEK293 cells were transfected with Ubi-Cas9 and WT-Cas9 plasmids and used for analysis. At 24 h following transfection, cells were treated with 50 μM cycloheximide (Sigma, A6185) at various time points from 0 to 24 h. After treatment, cells were harvested and lysed in ice-cold 1% Triton-X100 with protease inhibitor mixture (Roche) and PMSF. Total proteins were then extracted, and their concentration was determined using the Bradford assay (Pierce). Western blotting was performed with monoclonal anti-Cas9 (MAC133-clone 7A9, 1:2000 dilution) to determine the half-life (t1/2) of Cas9 protein, and anti–γ-tubulin (Sigma; 1:10000 dilution) was also probed to detect a loading control.

### PCR analysis of targeted embryos and single blastomere

We isolated single blastomere from 4–16 cell stage embryos for PCR. Zonae pellucidae of selected embryos were removed by exposure (20–30 sec) to Acid Tyrode’s solution. Zona free embryos were exposed (1–2 min) to 0.05% Trypsin to facilitate the separation of blastomeres and then transferred to PCR tube with 5 μl lysis buffer. Monkey embryos injected with Cas9/gRNA complex were collected and lysised (50 mM Tri-HCL(pH 8.5), 1 mM EDTA (ph8.0), 0.5% tween 20, 20 mg/L proteinase K) for PCR. The *Pink1, Parkin and ASPM* genes containing the target sites were determined by two rounds of PCR with nested primers for each one. The first round PCR was performed by initial incubation at 95 °C for 5 min, followed by 35 cycles of 95 °C for 30 s, 62 °C for 45 s, and 72 °C for 45 s. By using the products of the first round PCR as the templates, the second round PCR was performed by initial incubation at 95 °C for 5 min, followed by 35 cycles of 95 °C for 30 s, 62 °C for 30 s, and 72 °C for 30 s. The PCR products were analyzed by T7E1 assay or digested by special enzymes to detect the targeted DNA mutations. PCR products corresponding to genomic modifications were then subcloned for sequencing to verify the mutation sequences.

## Additional Information

**How to cite this article**: Tu, Z. *et al*. Promoting Cas9 degradation reduces mosaic mutations in non-human primate embryos. *Sci. Rep.* **7**, 42081; doi: 10.1038/srep42081 (2017).

**Publisher's note:** Springer Nature remains neutral with regard to jurisdictional claims in published maps and institutional affiliations.

## Supplementary Material

Supplemental Information
