# Supplementary material for: Promoting Cas9 degradation reduces mosaic mutations in non-human primate embryos
Source: Sci Rep. 2017 Feb 3;7:42081. doi: 10.1038/srep42081 (PMC5290468; doi:10.1038/srep42081)

Supplemental Information

Promoting Cas9 degradation reduces mosaic mutations in non-human primate embryos

Zhuchi Tu[1*], Weili Yang[1,2*], Sen Yan[2*], An Yin[1], Jinquan Gao[1], Xudong Liu[1], Yinghui Zheng[1], Jiezhao Zheng[3], Zhujun Li[3], Su Yang[4], Shihua Li[4], Xiangyu Guo[2#], Xiao-Jiang Li[1,2,4#]

[1]State Key Laboratory of Molecular Developmental Biology, Institute of Genetics and Developmental Biology, Chinese Academy of Sciences, Beijing, China 100101.

[2]GHM Institute of CNS Regeneration, Jinan University, Guangzhou 510631, China

[3]Yuanxi Biotech Inc., Guangzhou, China 510663.

[4]Department of Human Genetics, Emory University School of Medicine, Atlanta, GA 30322.

- * The first three authors contributed equally to this work.
- # Correspondence: xli2@emroy.edu (X.J.L.), guosapphire@yahoo.com (X.Y.G.)

Experimental Procedures

Preparation of Cas9 protein and in vitro activity assay. The Cas9 protein was prepared as described previously[39]. Briefly, the Pet-28b-cas9-His (plasmid#47327) plasmid encoding His-tagged Cas9 was expressed in E. coli, purified with Ni-NTA agarose resin (Qiagen), and eluted with 20 mM HEPES-KOH, pH 7.5, 150 mM KCl, 1 mM DTT, and 10% glycerol, followed by dialysis.

To validate the purified Cas9 activity in vitro, 4 nM PCR products containing the Pink1 or Parkin gene target sequences were mixed with purified His-Cas9 recombinant protein (WT-Cas9 and Ubi-Cas9) at different concentrations in NEB buffer 3 with corresponding gRNAs for the Pink1 and Parkin genes on ice for different times, as described previously [40]. The PCR products used for in vitro digestions were isolated with the following primers: monkey Pink1 Exon2 (sense 5'- CTCGCTGCACCTCTCTCTG-3' and antisense 5'-GGTGATCTGTGGGCAGAATA-3') and Parkin (sense 5'- GGGCAACTCTGTTTTTCACAA -3' and antisense 5'- TCCTGCTTGCTGTTTTAATGC -3').

Supplemental Figures

Figure S1. In Vitro Assays of Cas9 Activity. (A) His-tagged WT-Cas9 and Ubi-Cas9 were purified and their different amounts (μg) were examined by SDS-PAGE and Coomassie Brilliant Blue staining. Purified BSA (1 μg) served as a control. (**B**) Different amounts (ng) of purified WT-Cas9 and Ubi-Cas9 were incubated with Pink1-1 DNA with its gRNA (50 ng) for 30 min at 37°C. Control is incubation with no gRNA. Arrow indicates the intact Pink1-1 DNA. (**C**) The relative levels of the intact Pink1 DNA (% of the amount with no gRNA) after incubation for 30 min with different amounts of WT-Cas9 or Ubi-Cas9. (**D**) In vitro digestion of Pink1 and Parkin DNA (200 ng) by purified WT-Cas9 and Ubi-Cas9 at different time points (min). Arrows indicate the intact targeted DNAs.

Figure S2. The DNA Targeting Effect of Ubiquitin-Targeted Cas9. (A) PCR diagnosis of targeting effects of Ubi-Cas9 in transfected HEK293 cells. PCR products from 14 groups transfected cells for the targeted Pink1 gene and Parkin gene were digested by

T7E1 or BlpI. WT DNAs served as controls. Green arrows indicate mutated DNAs. (**B**) Sequence results of subcloned PCR products showing indel mutations in exon 2 of the Parkin gene and exon 2 of the Pink1 gene in HEK293 cells that were transfected with Ubi-Cas9 and respective gRNA plasmids. M: Molecular markers. The numbers of cloned DNA for sequencing and mutated DNA clones are indicated in parentheses (mutated clones/total clones).

Supplemental Reference

39 Cho, S. W., Kim, S., Kim, J. M. & Kim, J. S. Targeted genome engineering in human cells with the Cas9 RNA-guided endonuclease. *Nat. Biotechnol.* **31**, 230-232 (2013).

40 Shao, Y. *et al.* CRISPR/Cas-mediated genome editing in the rat via direct injection of one-cell embryos. *Nat. Protoc.* **9**, 2493-2512 (2014).

Supplemental Fig. 1

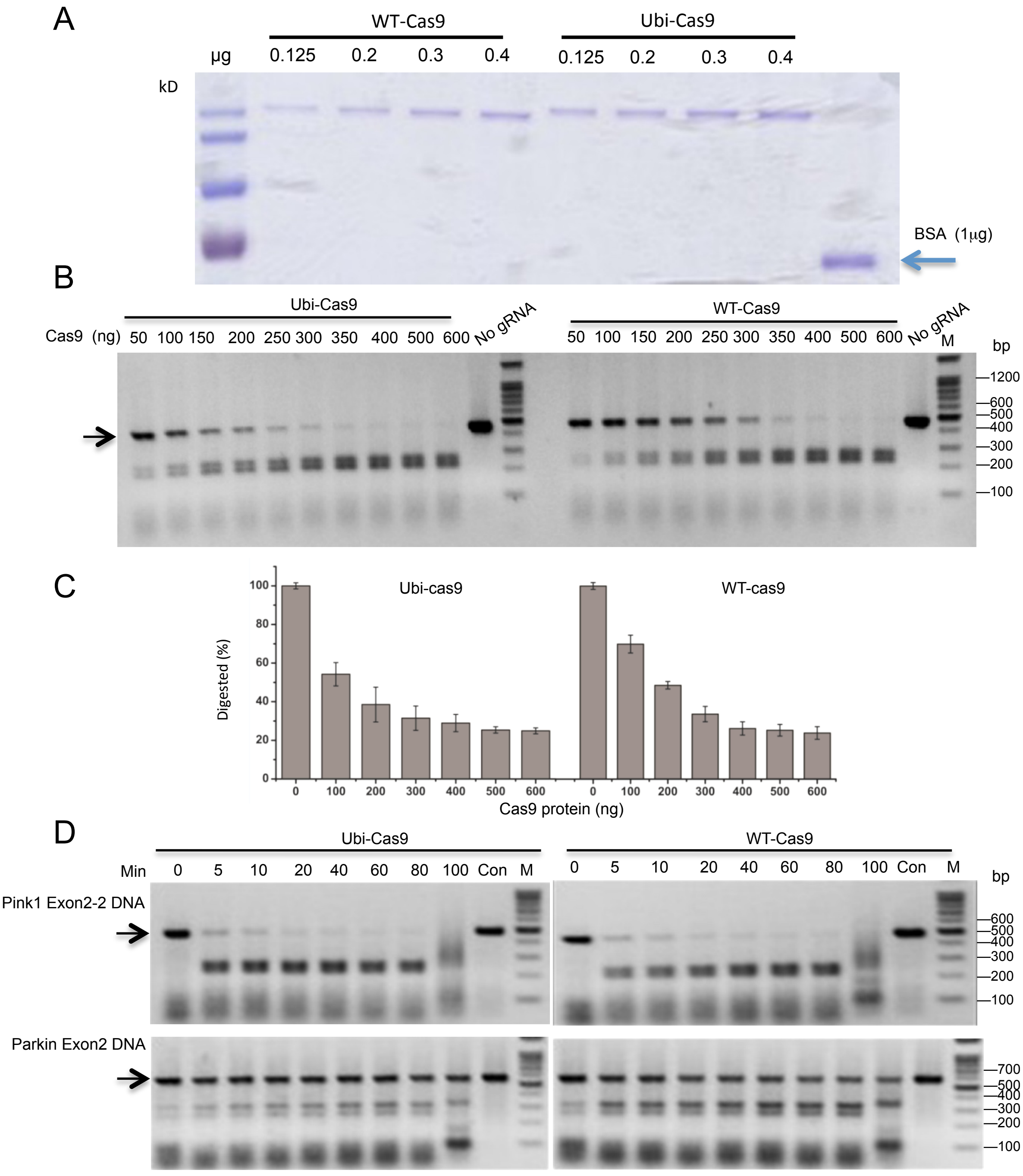

Supplemental Fig. 2

A

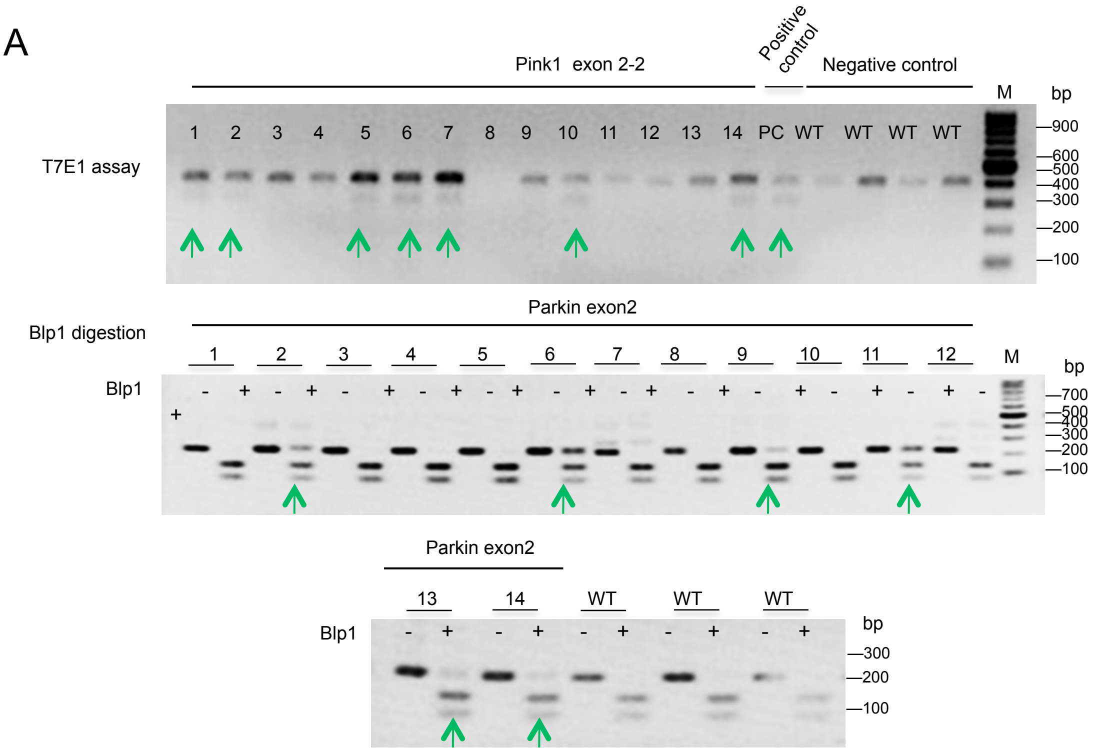

B

Pink1 exon2-2 WT GGCTTTCGGCTGGAGGAGTATCTGATAGGGCAGTCCATTGGTAAGGGCTGCAGTGCTGCT

Targeted GGCTTTCGGCTGGAGG---TAAGGGCTGCAGTGCTGCT -25bp （3/5）

Parkin exon2 WT TC TTCCAGCTCAAGGAGGTGGTTGCTAAGCGA CAGGGGGTTCCG GCTGACCAGTTG CGT

Targeted TCTTCCAGCTCAAGGAGGTGG-------CT---------------------------GTTCCGGCTGACCAGTTGCGTG -15bp (2/15)

TCTTCCAGCTCAAGGAGGGGGTT ----------------------AGGGGGTTCCGGCTGACCAGTTGCGTG -10bp (5/15)

TCTTCCAGCTCAAGGAGGTGGTTGCTAA-------GACAGGGGGTTCCGGCTGACCAGTTGCGTG -2bp (3/15)

Supplement material for Fig.1

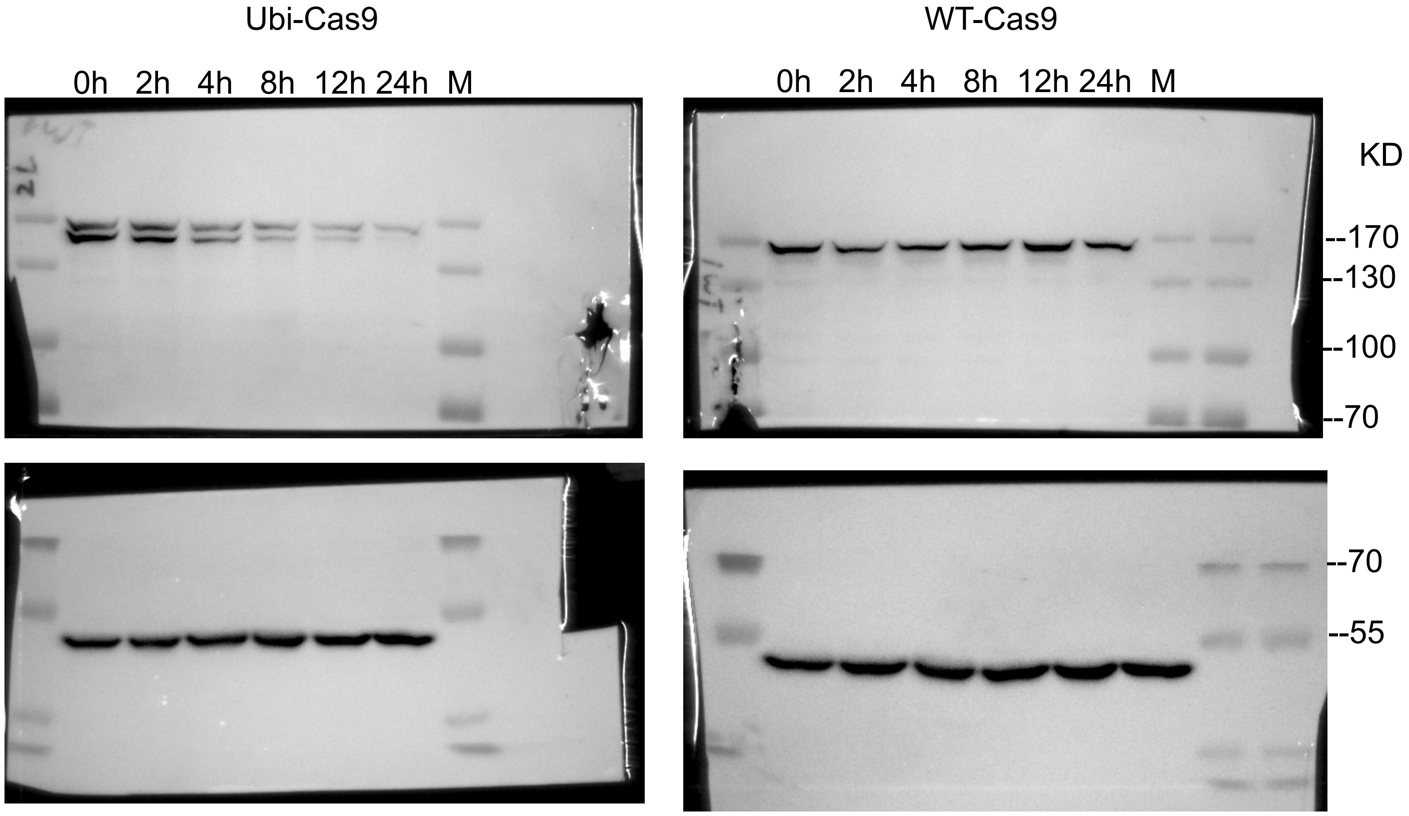

Supplement material for Fig.3

Pink 2-2 cut by Hpy1881

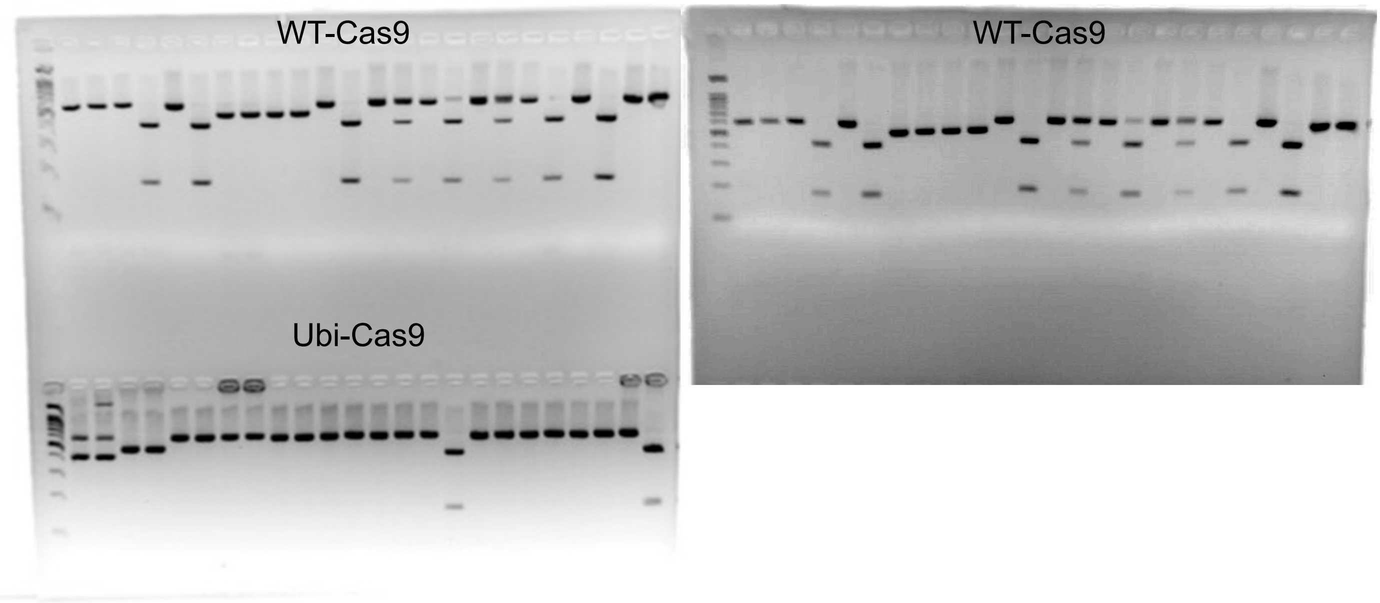

Supplement: Supplemental Information [file srep42081-s1.pdf]
